# The Effect of Capacitive and Resistive Electric Transfer Intervention on Delayed-Onset Muscle Soreness Induced by Eccentric Exercise

**DOI:** 10.3390/ijerph19095723

**Published:** 2022-05-08

**Authors:** Masatoshi Nakamura, Shigeru Sato, Ryosuke Kiyono, Kaoru Yahata, Riku Yoshida, Kazuki Kasahara, Andreas Konrad

**Affiliations:** 1 Faculty of Rehabilitation Sciences, Nishi Kyushu University, 4490-9 Ozaki, Kanzaki 842-8585, Saga, Japan; 2 Institute for Human Movement and Medical Sciences, Niigata University of Health and Welfare, 1398 Shimamicho, Kitaku 950-3198, Niigata, Japan; hpm19006@nuhw.ac.jp (S.S.); hpm19005@nuhw.ac.jp (R.K.); hpm20011@nuhw.ac.jp (K.Y.); hpm21017@nuhw.ac.jp (R.Y.); rpa18029@nuhw.ac.jp (K.K.); 3 Institute of Human Movement Science, Sport and Health, Graz University, Mozartgasse 14, 8010 Graz, Austria

**Keywords:** range of motion, maximum voluntary contraction, muscle damage, muscle pain

## Abstract

This study aimed to investigate the acute effect of capacitive and resistive electric transfer (CRet) intervention on eccentrically damaged muscle. A total of 28 healthy and sedentary male volunteers were randomly allocated to either CRet intervention or control groups. The participants performed a bout of eccentric exercise of the knee extensors with the dominant leg and received 30 min of CRet intervention of the quadriceps 48 h after the exercise. The dependent variables for the analysis were knee flexion range of motion (ROM), muscle soreness and maximum voluntary isometric (MVC-ISO), and concentric contraction (MVC-CON) torque of the knee extensors. These were measured prior to exercise (baseline) and before and after CRet intervention (48 h after the exercise). The results showed that knee flexion ROM, muscle strength (MVC-ISO and MVC-CON), and muscle soreness significantly improved after CRet intervention. CRet intervention may improve muscle soreness and loss of muscle function in an eccentrically damaged muscle.

## 1. Introduction

It is well known that resistance training emphasizing eccentric contraction (ECC) allows for more significant increases in muscle strength and muscle volume than resistance training emphasizing concentric contraction [1,2]. However, ECC has a negative impact, i.e., delayed-onset muscle soreness (DOMS), causing pain and muscle stiffness [3,4,5]. DOMS often occurs 24 h after intensive exercise (including ECC), peaking at 24–72 h and then subsiding within 5–7 days [6,7]. Since DOMS leads to decreased motivation for exercise and inhibits the continuation of exercise for a certain period, establishing its prevention and treatment methods is necessary [8].

A recent meta-analysis concluded that active recovery, massage, compression garments, immersion, contrast water therapy, and cryotherapy induce small to large decreases in DOMS magnitude; massage intervention seems to be the most effective approach [8]. Another meta-analysis investigating the effect of heat and cold therapy showed that both heat and cold therapy within 1 h after the exercise could reduce DOMS [9]. Together, massage and heat therapy could reduce DOMS and loss of muscle function, such as muscle strength and range of motion (ROM).

Most previous studies have investigated the preventive effect of interventions immediately after the exercise for DOMS; few have investigated treatments for DOMS 24–72 h after the exercise. Previous studies focusing on treatment effects showed that stretching interventions [10,11,12], foam rolling [13,14], and vibration foam rolling [15,16] could reduce the degree of pain in DOMS. However, to the best of our knowledge, no study investigated the effect of massage with the thermal agent on pain and loss of muscle function due to DOMS.

Capacitive and resistive electric transfer (CRet), a diathermy method for deep thermotherapy, has been recently developed [17]. CRet intervention consists of oscillating energy at specific frequencies generating therapeutic heat in body tissues [18]. Studies have shown that CRet can increase deep tissue temperature, whereas a hot pack only provides superficial thermotherapy [19], and can improve blood circulation in the muscle [20] and the peritendinous region [21]. CRet can simultaneously provide thermal stimulation and massage by moving the electrode. In a previous study, CRet intervention could promote recovery from fatigue in running tasks [22]. Additionally, CRet intervention could be useful for musculoskeletal disorders such as knee osteoarthritis [23] and chronic low back pain [24,25]. In addition, a systematic review of the CRet intervention concluded that CRet intervention could be a useful approach to decrease pain and improve the quality of life and disability of patients affected by musculoskeletal disorders [26]. Thus, it is hypothesized that the effect of CRet intervention on muscle damaged by ECC exercise is significant. This study aimed to investigate the acute effect of CRet intervention on DOMS and loss of muscle function, e.g., ROM and muscle strength in damaged muscle 48 h after ECC exercise.

## 2. Materials and Methods

### 2.1. Participants

A total of 28 healthy and sedentary male volunteers who had not performed any regular resistance or flexibility training for the past six months were recruited for this study. Participants without a history of neuromuscular disease or musculoskeletal injury of the lower extremity were included in this study. The participants were allocated randomly to either the CRet or control group using the alternation method. Thus, the CRet group had 15 participants (age, 21.5 ± 1.0 years; height, 170.9 ± 5.2 cm; body mass, 63.9 ± 6.5 kg), and the control group had 13 participants (age, 21.1 ± 0.3 years; height, 170.3 ± 5.2 cm; body mass, 62.2 ± 6.3 kg) (mean ± standard deviation [SD]). There were no significant differences in characteristics between groups using an unpaired *t*-test. All participants provided written informed consent. The study was approved by the relevant Ethics Committee (#18584) and complied with the requirements of the Declaration of Helsinki.

The sample size required for this study was estimated by G* power 3.1 software (Heinrich Heine University, Düsseldorf, Germany). The sample size for a split-plot analysis of variance (ANOVA) (alpha error = 0.05, power = 0.80, effect size = 0.40 [large]) was calculated, and more than 10 participants in each group is needed for this study. The large effect size was chosen based on the muscle soreness results of the previous studies [13,16].

### 2.2. Experimental Design 

**Figure 1:**
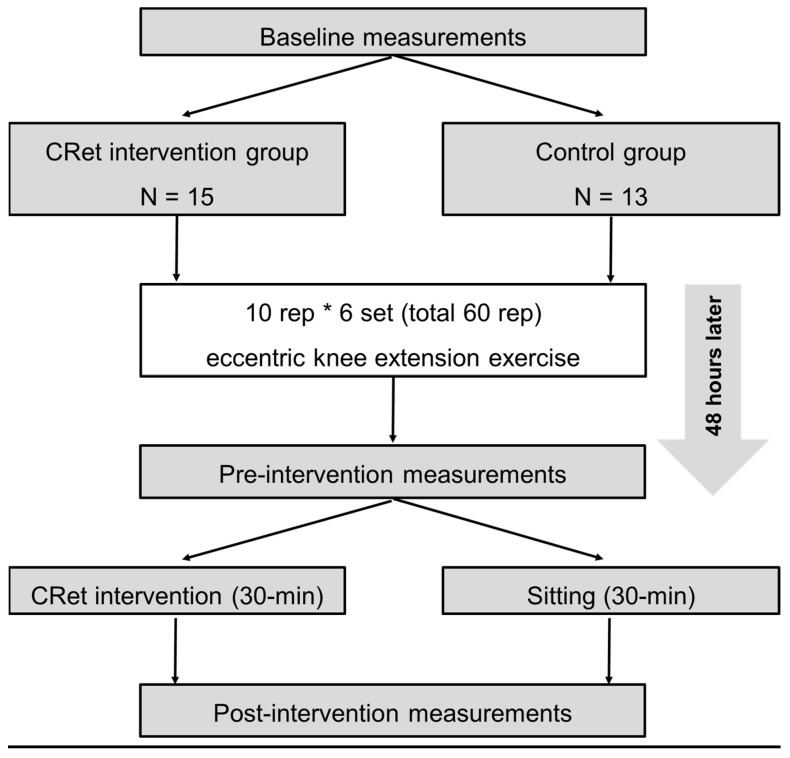
Experimental Design.

On the first day, outcome variables were measured before 60 repetitions of ECC exercises (baseline) [11,12,13]. Subsequently, the variables were measured 48 h after the ECC exercise (pre-intervention). After that, CRet group participants received 30 min of CRet intervention to the quadriceps (as described below). The control group participants sit on a chair for 30 min. The post-intervention measurements were performed immediately after CRet intervention or 30 min of sitting. The participants were familiarized with all measurements and ECC exercises before baseline measurement (Figure 1). A previous study confirmed the test–retest reliability of all outcome measurements for damaged muscles [13].

### 2.3. MVC-ISO and MVC-CON

**Figure 2:**
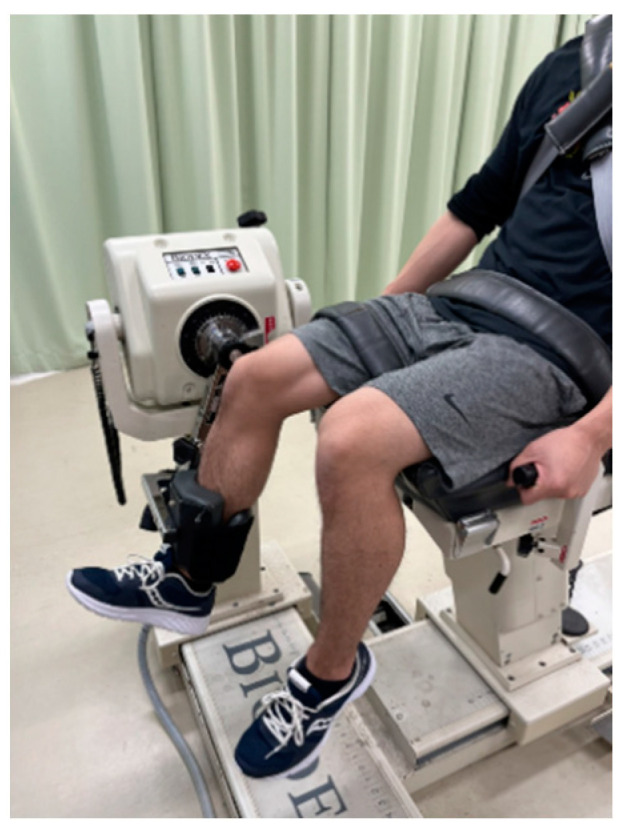
The set-up for knee extension muscle strength measurements.

Based on the previous studies [11,12,13], MVC-ISO was measured at two different knee angles (20° and 70°) using the isokinetic dynamometer (Biodex System 3.0, Biodex Medical Systems, Shirley, NY, USA) after correction for gravity. The participants sit in the dynamometer chair at a hip flexion angle of 80° with Velcro straps fixed over the exercised limb’s trunk, pelvis, and thigh (Figure 2). The knee joint of the dominant side was aligned with the axis of rotation of the dynamometer. The participants were instructed to perform maximal muscle contraction for 5 s at each angle, twice, with a 60 s rest between trials. The average value was adopted for further analysis.

MVC-CON was measured at the angular velocity of 60°/s for a 70° ROM (20°–90° knee angle) for 5 continuous maximal voluntary concentric contractions for both directions [11,13]. The highest obtained value in five trials was adopted for further analysis. Consistent verbal encouragement was provided during all tests.

### 2.4. Knee Flexion ROM

**Figure 3:**
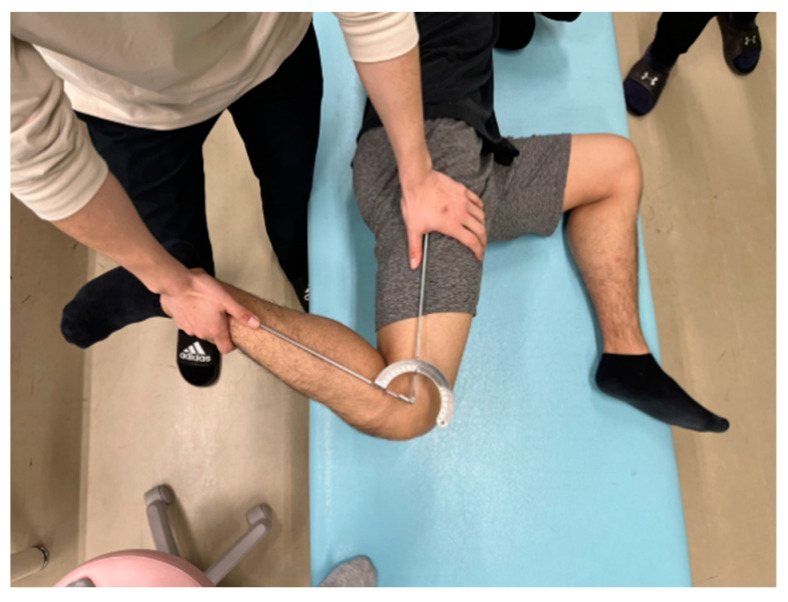
The knee flexion range of motion measurement.

The same physical therapist with more than ten years of experience measured the knee flexion ROM. Each participant was placed in a side-lying position on a massage bed; the hip and knee of the non-dominant leg were flexed at 90° to prevent the movement of the pelvis during measurement. The investigator brought the participant’s dominant leg to full knee flexion, with the hip joint in a neutral position (Figure 3). A goniometer (MMI universal goniometer Todai 300 mm, Muranaka Medical Instruments, Co., Ltd., Osaka, Japan) was used to measure knee flexion ROM three times, and the average value was used for further analysis [11,13].

### 2.5. Muscle Soreness

The magnitude of knee extensor muscle soreness was assessed for muscle contraction, stretching, and palpation using a visual analog scale consisting of a 100 mm line from “not sore at all” (0 mm) to “very, very sore” (100 mm) [27,28]. Muscle soreness at contraction was measured during both MVC-ISO and MVC-CON, and the average values were adopted for further analysis [13]. The same physical therapist with at least 10 years of experience measured muscle soreness during palpation when pressure was applied with the thumb. The participants laid supine on a massage bed, and the investigator palpated the proximal, middle, and distal points of the vastus medialis, vastus lateralis, and rectus femoris [11,13,29]. The location of the muscle soreness at palpation by palpation was identified based on the length of the thigh. The average values of the knee extensor palpation points were used for further analysis. In addition, muscle soreness at stretching was defined as muscle soreness during ROM measurement.

### 2.6. Eccentric Exercise Task

Using the dynamometer, all participants performed six sets of ten-repetition ECC exercises of the unilateral dominant leg [11,12,13]. As in previous studies [11,13], the participants were instructed to perform the maximal ECC from a slightly flexed position (20°) to a flexed position (110°) at an angular velocity of 60°/s. After each ECC, the lever arm passively returned the knee joint to the starting position (20° knee flexion) at 10°/s, i.e., gave a 9 s rest between each ECC contraction. Each exercise was repeated ten times for six sets, with a 100 s rest between sets. The participants received strong verbal encouragement to generate maximum force during each ECC exercise.

### 2.7. CRet Intervention

Indiba^®^ Active Pro Recovery HCR902 (Indiba, Barcelona, Spain) was used for CRet intervention in this study. This device operates at a frequency of 448 kHz. A rigid circular metallic electrode with a 65 mm diameter as the active electrode and a large flexible rectangular metallic plate (200 × 260 mm) as the inactive electrode was used in this study. Radiofrequency energy was delivered using either capacitive (CAP) or resistive (RES) mode from the active electrode. In the CRet group, the participants received 10 min of CAP-mode intervention and 20 min of RES-mode intervention. The active electrode was continually moved in a circular motion over the skin of the anterior thigh, with the inactive electrode placed under the thigh. A manufacturer-supplied conductive cream was used as a coupling medium between the active electrode and the skin surface. The heat intensity of the intervention was subjectively determined by a score of 6 or 7 on an 11-point analog scale for self-reporting of thermal sensation (0, no thermal sensation; 10, worst possible thermal sensation) to avoid burns. The intensity and duration of CRet intervention were based on the manufacturer’s recommendations, which were considered the most effective levels are not causing discomfort or pain [19,21]. 

### 2.8. Statistical Analysis

SPSS (version 24.0; SPSS Japan, Tokyo, Japan) was used for statistical analysis. The data for assumptions of normality were analyzed using a Shapiro–Wilk test. The differences between the CRet and control groups at baseline were assessed using the unpaired *t*-test. For all variables, a split-plot ANOVA was performed using the factors of time (baseline vs. pre-intervention vs. post-intervention) and group (CRet vs. control) to determine the interaction and main effects. Classification of effect size was set where η_p_^2^ < 0.01 was considered small, 0.02–0.1 was considered medium, and over 0.1 was considered to be a large effect size based on previous studies [30,31]. Significant differences between baseline, pre-intervention and post-intervention were determined using a Bonferroni post hoc test in each group when there were interaction effects. The effect size (Cohen’s d) was calculated as the difference in the mean value divided by the pooled SD between pre- and post-intervention in the CRet group [16]. A Cohen’s d value of 0.00–0.19 was considered trivial, 0.20–0.49 as small, 0.50–0.79 as moderate, and ≥0.80 as large [30,32]. The difference was considered to be statistically significant at an alpha level of *p* < 0.05.

The relationship between changes from baseline to pre-intervention and from pre-intervention to post-intervention in muscle soreness during muscle contraction, stretching, and palpation was investigated. This was also used to quantify the relationship between relative changes (%) from baseline to pre-intervention and from pre-intervention to post-intervention in MVC-ISO, MVC-CON, and ROM. Pearson’s correlation analysis was used since the data were normally distributed. The absolute value of correlation coefficient (r) can be interpreted as |r| ≤ 0.1 was no correlation, 0.1 < |r| ≤ 0.3 was mild/modest correlation, 0.3 < |r| ≤ 0.6 was moderate correlation, 0.6 < |r| < 1 was strong correlation, and |r| = 1 was perfect correlation [33]. Data are presented as mean ± SD.

## 3. Results

Table 1 presents all variables in both CRet and control groups. Split-plot ANOVA showed significant interactions for MVC-ISO (*p* < 0.01; F = 5.44; η_p_^2^ = 0.179), MVC-CON (*p* = 0.014; F = 4.70; η_p_^2^ = 0.158), and knee flexion ROM (*p* = 0.02; F = 4.21; η_p_^2^ = 0.144). Post hoc testing in the CRet group showed that MVC-ISO, MVC-CON, and knee flexion ROM were significantly decreased 48 h after ECC exercise (pre-intervention), compared with baseline values (*p* < 0.01), but were significantly (*p* < 0.01) recovered post-intervention (d = 0.58, d = 0.96, and d = 1.23, respectively). In the control group, MVC-ISO, MVC-CON, and knee flexion ROM were significantly decreased 48 h after ECC exercise (pre-intervention), compared with baseline values, but did not recover post-intervention, except for MVC-ISO.

Split-plot ANOVA showed no significant interactions for muscle soreness for contraction (*p* = 0.195; F = 1.69; η_p_^2^ = 0.063) and stretching (*p* = 0.166; F = 1.86; η_p_^2^ = 0.069) but showed main effects for time (*p* < 0.01; F = 22.0; η_p_^2^ = 0. 468 and *p* < 0.01; F = 49.5; η_p_^2^ = 0.664, respectively). Post hoc testing showed that muscle soreness for contraction at pre-intervention and post-intervention was significantly higher than at baseline and that values at post-intervention were significantly lower than at pre-intervention. In addition, muscle soreness for stretching at pre-intervention was significantly higher than at baseline and post-intervention. Conversely, split-plot ANOVA showed a significant interaction for muscle soreness at contraction (*p* < 0.01; F = 5.71; η_p_^2^ = 0.186). 

In the CRet group, post hoc testing showed that muscle soreness for palpation was significantly increased 48 h after ECC exercise, compared with baseline values (*p* < 0.01), but significantly recovered post-intervention (*p* < 0.01, d = 1.14). However, in the control group, muscle soreness for palpation significantly increased 48 h after ECC exercise (*p* < 0.01), compared with baseline values, but did not recover post-intervention (*p* < 0.01).

**Figure 4:**
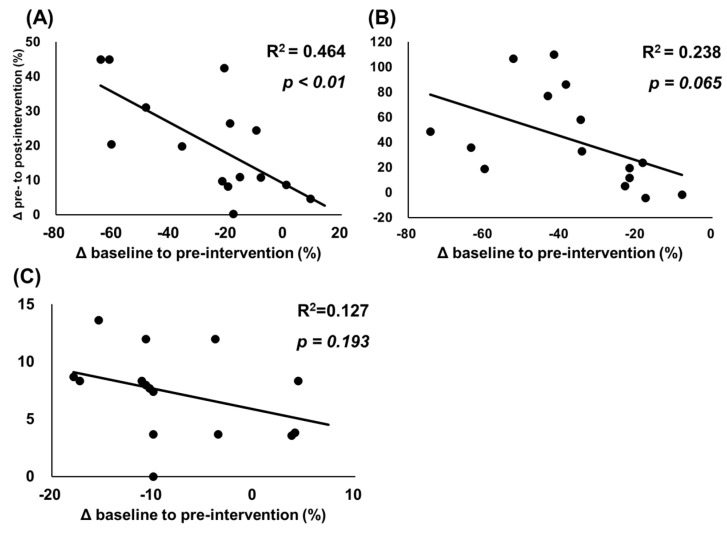
Relationships between relative changes in muscle strength and range of motion. Data presented as individual data points with R^2^ and *p* values. The figure shows relationships between relative changes (%) from baseline to pre-intervention and from pre-intervention to post-intervention in maximal voluntary isometric contraction (**A**), maximum voluntary concentric contraction of the knee extensors (**B**), and passive knee flexion range of motion (**C**).

**Figure 5:**
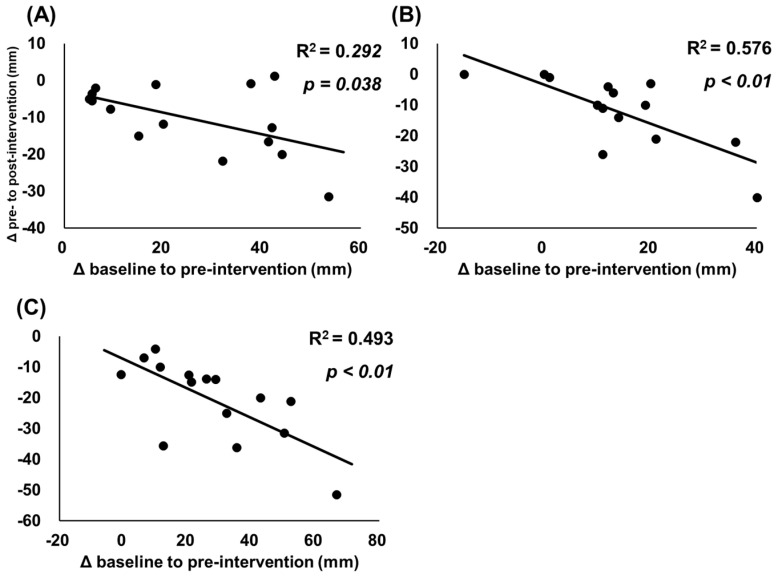
Relationships between changes in muscle soreness. Data presented as individual data points with R^2^ and *p* values. The figure shows relationships between changes from baseline to pre-intervention and from pre-intervention to post-intervention in muscle soreness for muscle contraction (**A**), stretching (**B**), and palpation (**C**).

The associations between changes from baseline to pre-intervention and from pre-intervention to post-intervention are shown in Figure 4 and Figure 5. Figure 4 shows significant negative associations for relative change (%) in MVC-ISO (r = 0.681, R^2^ = 0.464, *p* < 0.01); however, there were no significant associations for and MVC-CON (r = −0.488, R^2^ = 0.238, *p* = 0.065) and knee flexion ROM (r = −0.356, R^2^ = 0.127, *p* = 0.193). Additionally, Figure 5 shows that significant negative associations were found between change (mm) in muscle soreness at muscle contraction (r = −0.540, R^2^ = 0.292, *p* = 0.038), stretching (r = −0.759, R^2^ = 0.576, *p* < 0.01), and palpation (r = −0.702, R^2^ = 0.493, *p* < 0.01).

## 4. Discussion

This study investigated the acute effect of a CRet intervention 48 h after ECC exercise on DOMS and loss of muscle function (muscle strength and flexibility). The results showed the following findings: (1) CRet intervention can promote the recovery of loss of MVC-ISO, MVC-CON, and knee flexion ROM since there were no significant differences in these measurements before ECC exercise and after CRet intervention; (2) CRet intervention can decrease muscle soreness at palpation, although there were no significant improvements in muscle soreness for contraction and stretching after CRet intervention, compared with the control group; (3) the effects of CRet intervention were significantly greater in participants with more severe DOMS and loss of muscle function. To the best of our knowledge, this is the first study to investigate the effect of CRet intervention on eccentrically damaged muscle.

Our results show that muscle strength decreased significantly after ECC exercise after CRet intervention; statistically, no difference from baseline values (i.e., before ECC exercise) was observed. A previous study investigating the effects of foam rolling or vibration foam rolling on eccentrically damaged muscle showed that muscle strength improved with this intervention, but not to the baseline values [13,15,16]. Therefore, it is clear that CRet intervention is a more effective recovery approach for eccentrically damaged muscles than foam rolling. Interestingly, muscle soreness for contraction significantly recovered after CRet intervention, compared with the control group, although no significant interaction effect was observed. Previous studies have observed improvements in muscle soreness can increase muscle strength after static stretching [10] and foam rolling interventions [13]. Therefore, our results suggest that CRet intervention could improve muscle strength. In addition, Tashiro et al. (2017) have shown that CRet intervention increases muscle temperature. Previous studies have reported that increased muscle temperature leads to increased performance [34,35]. Therefore, it is possible that CRet intervention may improve muscle strength because of pain relief and increased muscle temperature.

CRet intervention significantly improved knee flexion ROM and increased muscle strength. Yokota et al. (2018) showed that CRet intervention could increase muscle flexibility, which is believed to have increased knee flexion ROM in this study. Although no significant interaction effect was observed, CRet intervention improved muscle soreness at stretching. Therefore, the decrease in muscle soreness at stretching due to CRet intervention may also be related to increased knee flexion ROM.

Muscle soreness at palpation was significantly improved by CRet intervention to a value that was not significantly different from the baseline value. Tashiro et al. (2020) investigated the effect of CRet intervention on patients with non-specific chronic low back pain. They showed that CRet intervention combined with exercise could decrease low back pain [24], consistent with our results. The mechanism of pain reduction after CRet intervention has not been fully clarified. A proposed mechanism is that thermal stimulation, directly and indirectly, affects pain reduction by improving ischemia and muscle spasticity [36,37]. Since an increase in afferent input from the A-β fibers can inhibit the effects of pain stimuli at the cord level, an increase in pain threshold may be at least partially due to thermal stimulation. Previous studies have shown that thermal stimulation can increase the pain threshold, as thermal stimulation of the nociceptive fibers (A-delta and C) may block pain transmission. In addition, the stimulation of temperature receptors increases vasodilation, alleviating pain due to ischemia. Ischemic pain is due to spasticity, which compresses blood vessels. CRet intervention may increase muscle temperature and blood flow more effectively than hot pack application [20]. The increase in muscle temperature and blood flow could be sustained for at least 30 min after intervention [20]. Therefore, improving pain is greater than that of the hot pack application and could last for a longer period.

Our study has shown significant negative correlations between muscle soreness and decreased muscle strength after ECC exercise and the improving effects of CRet intervention (Figure 4 and Figure 5). Our results suggest that participants with muscle soreness or muscle function loss after ECC exercise could greatly improve after CRet intervention, which is consistent with a previous study investigating the effect of a foam rolling intervention on eccentrically muscle damage [13]. In terms of clinical application for sports and rehabilitation settings, CRet is a practical treatment approach for painful muscles, especially for participants with pain and a great loss of muscle function.

Previous studies focusing on treatment effects showed that stretching interventions [10,11,12], foam rolling [13,14], and vibration foam rolling [15,16] could reduce the degree of pain in DOMS. However, these interventions can be painful and thus place a high burden on the participants. On the other hand, the CRet intervention is less painful and effective in improving the loss of muscle function and muscle soreness in DOMS. In addition, the previous study showed that CRet intervention could promote recovery from fatigue in running tasks [22]. Additionally, a systematic review showed that a CRet intervention could increase skin temperature, enhance skin and muscle blood perfusion, as well as report an increase in oxyhemoglobin [18]. Thus, a CRet intervention could promote the recovery from damaged muscle as well as an immediate effect. In addition, a CRet intervention could be useful for musculoskeletal disorders such as knee osteoarthritis [23] and chronic low back pain [24,25]. In addition, a systematic review of the CRet intervention concluded that CRet intervention could be a valuable approach to decrease pain and improve the quality of life and disability of patients affected by musculoskeletal disorders [26]. Therefore, CRet intervention is expected to be applied in sports and rehabilitation fields.

The present study has some limitations. First, since only the acute effect of CRet intervention was investigated in this study, the sustained and recovery effects of CRet intervention remain unclear. Second, this study did not investigate the sham/placebo intervention and did not compare the CRet and hot pack interventions for eccentrically damaged muscle. Third, the participants were not athletes but sedentary male volunteers. Forth, this study investigated only muscle strength and muscle soreness, and the effect of CRet intervention on dynamic muscle performance, such as jump and sprint performance, remains unclear. Thus, a future study is needed to apply CRet intervention in sports and rehabilitation.

## 5. Conclusions

This study investigated the acute effect of CRet intervention on damaged muscle after 48 h of ECC exercise and showed that CRet intervention is an effective approach for improving muscle soreness and loss of muscle function (decreases in muscle strength and flexibility). The improvement effect was greater in participants with greater muscle soreness and more decreased muscle function. Therefore, CRet intervention can be recommended as a treatment for muscle soreness and associated muscle dysfunction.

## Figures and Tables

**Table 1:** The changes in maximum voluntary isometric contraction (MVC-ISO), maximum voluntary concentric contraction (MVC-CON) torque of the knee extensors, knee flexion range of motion (ROM), and muscle soreness at contraction, stretching, and palpation in capacitive and resistive electric transfer (CRet) intervention and control groups.

		Baseline	Pre-Intervention	Post-Intervention	Interaction Effect
MVC-ISO (Nm)	CRet group	154.3 ± 23.1	112.1 ± 34.1 *	132.0 ± 34.8 ^#^	F = 5.44, *p* < 0.01
	Control group	162.1 ± 22.9	98.8 ± 43.7 *	100.0 ± 34.5 *	η_p_^2^ = 0.179
MVC-CON (Nm)	CRet group	164.9 ± 26.7	103.6 ± 34.5 *	141.5 ± 44.7 ^#^	F = 4.70, *p* = 0.014
	Control group	154.0 ± 19.8	82.3 ± 34.3 *	91.8 ± 34.7 *^,#^	η_p_^2^ = 0.158
Knee flexion ROM (°)	CRet group	138.0 ± 9.6	126.7 ± 8.3 *	135.7 ± 6.3 ^#^	F = 4.21, *p* = 0.02
	Control group	143.2 ± 8.5	136.8 ± 10.9 *	138.3 ± 10.8 *	η_p_^2^ = 0.144
Muscle soreness					
At contraction (mm)	CRet group	2.2 ± 5.4	27.4 ± 15.3	17.2 ± 13.4	F = 1.69, *p* = 0.195
	Control group	4.9 ± 4.2	24.4 ± 12.8	23.0 ± 11.5	η_p_^2^ = 0.063
At stretching (mm)	CRet group	25.9 ± 9.1	39.4 ± 15.1	27.5 ± 13.0	F = 1.86, *p* = 0.17
	Control group	34.8 ± 25.0	49.8 ± 25.2	44.3 ± 26.6	η_p_^2^ = 0.069
At palpation (mm)	CRet group	14.7 ± 10.3	45.7 ± 17.3 *	21.5 ± 14.6 ^#^	F = 5.71, *p* < 0.01
	Control group	14.7 ± 10.3	45.7 ± 17.3 *	40.4 ± 11.7 *	η_p_^2^ = 0.186

*: significant difference from baseline value; ^#^: significant difference from pre-intervention value. Statistical differences fixed at *p* < 0.05.

## Data Availability

The raw data supporting the conclusions of this article will be made available by the authors without undue reservation.
